# Mutation Profile via Next-Generation Sequencing in Patients with Colorectal Adenocarcinoma and Its Clinicopathological Correlation

**DOI:** 10.5152/tjg.2023.22682

**Published:** 2023-11-01

**Authors:** Sibel Osman, Nesibe Kahraman Çetin, İbrahim Halil Erdoğdu, İbrahim Meteoğlu

**Affiliations:** Department of Pathology, Aydın Adnan Menderes University Faculty of Medicine, Aydın, Turkey

**Keywords:** Colorectal cancer, molecular, mutation, NGS

## Abstract

**Background/Aims:**

Recent studies that reveal the molecular profiles of colorectal carcinomas have demonstrated tumor heterogeneity. Characterization of colorectal carcinoma-specific genomic alterations is essential for developing more successful and targeted treatment protocols. Moreover, it is vital in elucidating the pathogenesis and mechanisms of resistance against treatment and predicting prognosis.

**Materials and Methods:**

The study included 73 cases diagnosed with colorectal carcinomas and subjected to molecular analysis by the next-generation sequencing. The association between the clinicopathologic parameters and pathogenic mutations detected in 32 genes was evaluated.

**Results:**

Pathogenic mutations were determined in a total of 24 genes. The Cell Division Cycle 27 (CDC27), Kirsten rat sarcoma viral proto-oncogene (KRAS), serine/threonine protein kinase B-raf (BRAF), phosphatase and tensin homolog, breast cancer 2 (BRCA2), and phosphotidylinositol-4,5-biphosphate 3-kinase (PIK3CA) mutations were determined at higher rates, with the adenomatous polyposis coli mutation determined at a lower rate than in the literature. There were significant positive correlations between CDC27 and phosphatase and tensin homolog (PTEN), PTEN and BRCA2, and PTEN and adenomatous polyposis coli (APC) concomitant mutations, whereas negative correlations were present between BRAF and KRAS. Statistically significant relationships were present between KRAS exon 2 and mucinous morphology, PIK3CA and absence of perineural invasion, BRAF and tumor differentiation/localization, MutS homolog 3 (MSH3) and tumor diameter, and BRCA2 and absence of lymph node metastasis.

**Conclusion:**

It is necessary to have a comprehensive database of genomic alterations of colorectal carcinomas to interpret mutations more accurately clinically. There are no studies on the frequency of mutations in colorectal carcinomas in the Turkish population; thus, follow-up and treatment protocols are organized following the European and American databases and guidelines. A comprehensive study of the colorectal carcinoma patients’ mutation profile in the Turkish patient cohort by the next-generation sequencing method will help to provide significant therapeutic, prognostic, and predictive data and design more successful treatment and follow-up strategies.

**Main Points**

- This is the first study conducted in the Turkish population to investigate the somatic mutation profile determined by the next-generation sequencing system in patients with colorectal carcinomas (CRCs) belonging to a specific region and their associations with the clinicopathologic parameters.
- We report that we detected the cell division cycle 27 (CDC27), Kirsten rat sarcoma viral proto-oncogene (KRAS), serine/threonine protein kinase B-raf (BRAF), phosphatase and tensin homolog (PTEN), breast cancer 2 (BRCA2), and phosphotidylinositol-4,5-biphosphate 3-kinase (PIK3CA) mutations at a higher rate and the adenomatous polyposis coli (APC) mutation at a lower rate than in the literature.
- We report how the significant positive correlations between CDC27 and PTEN, PTEN and BRCA2, and PTEN and APC concomitant mutations and the significant negative correlation between BRAF and KRAS mutations are associated with tumor development and progression and increased resistance or sensitivity against treatments.
- We contribute to the literature by reporting the significant relationships between KRAS exon 2 and mucinous morphology, PIK3CA and absence of perineural invasion, BRAF and tumor differentiation and localization, MutS homolog 3 (MSH3) and tumor diameter, and BRCA2 and absence of lymph node metastasis.

## Introduction

Colorectal carcinoma (CRC) is not a homogeneous disease, and identifying different molecular pathways in its carcinogenesis has revealed its heterogeneous nature.^1,2^ Understanding the molecular basis of colorectal carcinogenesis is of utmost importance for both the prognosis and treatment of these tumors.^1^ Molecular tests play a role not only in determining the treatment protocol but also in understanding the resistance mechanisms against treatment, predicting the disease prognosis, and clarifying the cancer pathogenesis.^1,2^ The data on the mutation profile of CRC and its relationship with clinicopathologic parameters are insufficient in our country. Therefore, collecting molecular data from our population is essential for correlating them with clinical and pathological data, predicting prognosis, avoiding unnecessary drug consumption by determining the mutations manifesting drug-specific resistance, determining effective treatment strategies, and improving treatment success and survival. The next-generation sequencing (NGS) method, which offers a comprehensive mutation profile, enables simultaneous analysis of multiple genes and regions within the genes. Thus, important information related to the patient’s clinical management can be obtained in the most economical way regarding time, cost, and tumor tissue. This study aimed to determine the profile of somatic mutations identified by the NGS system and compare them with the clinicopathologic parameters in CRC patients. Unfortunately, the number of studies in this field is very limited in the literature. By revealing the molecular changes specific to our population, this study will guide individualized treatment and contribute significantly to determining the prognosis.

## Materials and Methods

The study was approved by the Aydın Adnan Menderes University Faculty of Medicine Non-Interventional Clinical Research Ethics Committee, Aydın, Turkey (number: 168/2020, date: 09/08/2022).

### Patients

This study included 73 cases diagnosed with adenocarcinoma in whom a colorectal panel involving 32 genes was studied through the NGS system in the Molecular Pathology Laboratory using the resection materials of colon and rectum examined in the Medical Pathology Department of Aydın Adnan Menderes University Faculty of Medicine between 2018 and 2020. The hematoxylin and eosin-stained preparations and blocks of the cases were removed from the archive and reevaluated with a light microscope (Olympus BX53, Olympus Co., Tokyo, Japan). In addition, the clinical features of the patients were obtained from the file records. The patient’s sex and age, the tumor localization, differentiation, diameter, pathological stage, lymphovascular and perineural invasion status, lymph node metastasis status, and analysis results of 32 cancer-related genes in the colorectal panel of the NGS system (Table 1) were recorded.

### Next-Generation Sequencing Study Method

The cancer panel’s routine clinical laboratory implementation consists of isolating genomic and free tumor deoxyribonucleic acid (DNA), target site enrichment in DNAs of appropriate quality and quantity, establishing study libraries, and NGS. Then, the study’s quality is determined by data analysis, and in line with the patient’s clinical history, the variants’ bioinformatic interpretations are made through variant analysis.

### Deoxyribonucleic Acid Extraction

The pathologist marked the tumor sites, and DNA was extracted from sections of 10 µm thickness using the Qiagen formalin-fixed paraffin-embedded DNA tissue extraction kit, following the manufacturer’s instructions. Deoxyribonucleic acid was quantified using a Qubit 3.0 dsDNA HS assay kit (Life Technologies, San Diego, California, USA) and Qubit fluorometer. The study continued with patients from whom 100-150 ng DNA was obtained.

### Preparing the Next-Generation Sequencing Library and Sequencing

This step was accomplished on the MiniSEQ NGS platform (MiniSEQ, MN00676, Illumina, Singapore) for the QIAseq targeted column DNA panel (DHS-002Z-12, Qiagen, Strasse, Hilden, Germany; Table 1). Formalin-fixed paraffin-embedded DNA fragments of 100-150 ng were subjected to end repair. Target enrichment was amplified via polymerase chain reaction (PCR; Labcycler, Sensoquest GmbH, Göttinger, Germany). Barcoding and library preparations were then performed. The target-enriched libraries were then sequenced on MiniSEQ NGS platforms (MiniSEQ) using a MiniSEQ High-Output Reagent Cartridge (Illumina, Inc., San Diego, Calif, USA).

### Data Analysis

Data analysis and quality control of sequencing results were performed using the universal commercial software Qiagen Clinical Insight Analysis. Once data quality was examined, the variants were imported into the Qiagen Clinical Insight Interpretation web interface that interprets the predefined variants’ data. The selected variants were analyzed using bioinformatics tools such as CADD (v1.3), Clinical trials (Stepford 181112.001), Allele Frequency Community, JASPAR, EVS (ESP6500SI-V2), Vista enhancer hg18, hg19, PolyPhen-2, Refseq gene model, and 1000 genome frequency (phase3v5b) to validate the diagnosis and evaluate their impact on clinical status and treatment protocols. Selected variations were identified using the Qiagen Clinical Insight Browser platforms and Qiagen reporter. A report consisting of a per-sample summary of findings was generated for each identified variable, followed by a direct link to the data source and the Qiagen Knowledge Base with the recommended treatment listed. The cutoff value for this panel’s detection was set at 5%, and the process of NGS took approximately 7 days.

### Statistical Analysis

Statistical analyses were performed with the Statistical Package for the Social Sciences 22.0 Software Package (IBM Corp.; Armonk, NY, USA). The differences regarding frequencies between the groups were evaluated using chi-square or Fisher’s tests (when the values observed in the cells did not meet the assumptions of the chi-square test). The result was considered statistically significant when the *P*-value was less than .05.

## Results

The clinicopathologic features of the 73 patients included in the study are summarized in Table 2.

### Mutation Profiles Detected via Next-Generation Sequencing and Their Relationships

**Figure 1.**
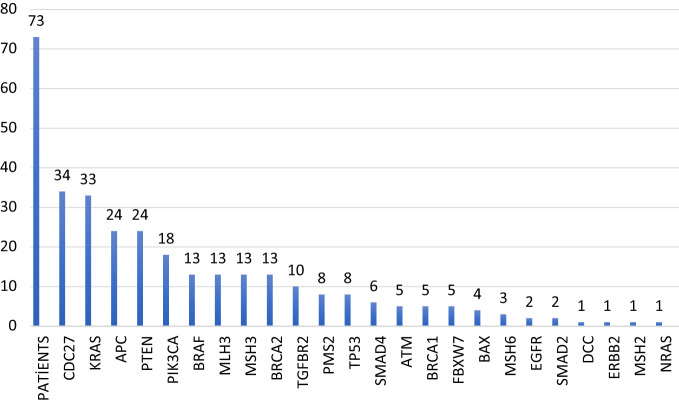
Distribution of mutations detected in our study.

In the NGS system, the variants determined in cancer-related genes are evaluated in 3 categories according to the guidelines of Association for Molecular Pathology/American Society of Clinical Oncology/College of American Pathologists.^3^ In this study, variants of strong significance (tier 1A and 1B), categorized as validated predictive and prognostic variants with well-defined cancer-associated genomic alterations in the database and/or literature, were included. Mutations were detected in 24 of the CRC panel’s 32 genes, and the mutations in our study are shown in Figure 1.

Twenty-six (78.8%) of the Kirsten rat sarcoma viral proto-oncogene (KRAS) mutations determined in 33 patients were in exon 2 (20 in codon 12 and 6 in codon 13), 4 (12.1%) in exon 3 (3 in codon 61 and 1 in codon 59), and 3 (9.1%) in exon 4 (2 in codon 146 and 1 in codon 117). In addition, mutations in KRAS exon 2 were identified in 7 (77.8%) of 9 tumors with mucinous morphology. Ten (76.9%) of serine/threonine protein kinase B-raf (BRAF) mutations identified in 13 patients (17.8%) were BRAF V600E, and 3 (23.1%) were non-BRAF V600E mutations.

All of the patients had more than 1 mutation. The concomitant mutations determined in our study are presented in Table 3. A statistically significant positive relationship was identified in cell division cycle 27 (CDC27)–adenomatous polyposis coli (APC), CDC27–phosphatase and tensin homolog (PTEN), CDC27–breast cancer 2 (BRCA2), CDC27–MutL homolog 3 (MLH3), CDC27–MutS homolog 3 (MSH3), APC–PTEN, APC–MLH3, APC–BRCA2, PTEN–MLH3, PTEN–MSH3, and PTEN–BRCA2 concomitant mutations (*P* < .05). None of the 13 patients with a BRAF mutation had a concurrent KRAS mutation. A statistically significant negative relationship was present between BRAF and KRAS mutations (*P* < .001).

### The Relationships Between Mutations and Clinicopathologic Parameters

A statistically significant positive relationship was present between mucinous morphology and KRAS exon 2 mutations (*P* = .042). Mutations in KRAS exon 3 were determined in 3 (27.3%) of 11 well-differentiated tumors. A statistically significant positive association was determined between KRAS exon 3 mutation and well-differentiated tumors (*P* = .028). Moreover, statistically significant positive correlations were present between stage pT2 tumors and the PTEN mutation (*P* = .048), proximal colon as the tumor localization and the BRAF V600E mutation (*P* < .01), and low differentiated tumors and the BRAF mutation (*P* = .025).

In our study, the optimal cutoff value of tumor diameter determined by receiver operating characteristic analysis was 4.35 cm. The number of patients with a tumor diameter of more than 4.35 cm was 37 (50.7%), and the MSH3 mutation was present in 10 of them (27%). A statistically significant positive correlation was found between MSH3 mutation and tumor diameter (*P* < .05). On the other hand, there were statistically significant negative correlations between perineural invasion and phosphotidylinositol-4,5-biphosphate 3-kinase (PIK3CA) mutation (*P* = .043) and between lymph node metastasis and BRCA2 mutation (*P* = .009).

## Discussion

Colorectal carcinoma is a remarkably heterogeneous disease with diverse clinical and molecular features. With NGS technology, our knowledge of mutations involved in colorectal carcinogenesis and the genomic profiles of CRCs is increasing with each passing day, leading to a better understanding of the tumor heterogeneity of CRCs.^1^ On the other hand, predicting responses to existing treatment options and developing new comprehensive and individualized treatments are only possible by resolving the ambiguity in the genomic profiles of tumors.^2^

Our study compared the somatic mutations in CRC identified by the NGS method to the literature data. Compared to the previous international^4-11^ and national^12,13^ data, some mutation rates are observed to have differences at the regional level (Table 4). At this point, it is imperative to mention some of the features of the NGS technique we used to detect somatic mutations in cancers that may lead to results similar to those in our study.^14^ With increasing sequenced genome area, the likelihood of finding rare and novel variants that need to be interpreted increases. When a variant is spotted, it is critical to confirm whether it represents an actual pathogenic event. Besides, from a medical point of view, since the pathogenic changes might be incidentally discovered by the NGS method, it is also essential to know which findings should be reported to the clinician. For this reason, guidelines about elucidating the pathogenicity of newly discovered variants have been published.^15^ It is necessary to interpret the data obtained after sequencing and define the actual variants with clinical information in consideration.^14^

Various studies have reported the CDC27 mutation rate in CDCs as over 5%, and this mutation is known to be associated with tumor proliferation and progression in CRCs.^4^ In our study, this somatic mutation was determined with the highest rate of 46.6%, which suggests regional differences. Even though some studies have suggested that CDC27 promoted metastasis development in CRCs^16^ and might be a potential prognostic indicator^4,17^ and a therapeutic target,^4,16,17^ there is insufficient literature data on its association with other mutations involved in colorectal carcinogenesis.^17^ CDC27 mutations, which our study found significantly associated with APC, PTEN, MLH3, MSH3, and BRCA2 mutations, seem to play an important role in CRC carcinogenesis. Considering that CDC27 is significantly phosphorylated during mitosis based on limited studies in the literature, PTEN has been suggested to be a potential phosphatase for CDC27. Furthermore, since the tumors with mutant PTEN together with CDC27 have been claimed to be less sensitive to treatments targeting the Anaphase Promoting Complex/Cyclosome-cdc20 Homolog 1 (APC/C-CDH1) complex, it should also be considered clinically relevant.^18^ It is evident that more advanced clinical studies are needed to determine at what stage and how these mutations are associated with carcinogenesis and to identify their effects on clinical and pathological features.

The KRAS mutation is highly prevalent in CRCs, with a rate of approximately 40%. The presence of KRAS mutation is considered an essential factor in the management of prognosis and treatment because it is a predictor of poor prognosis and an indicator of non-response to epidermal growth factor receptor (EGFR)-targeted treatments.^5,19^ Our study’s KRAS mutation rate was 45.2%, which is higher than the literature average. About 90% of KRAS mutations occur in exon 2, codons 12 and 13; roughly two-thirds of these mutations are in codon 12 and one-third are in codon 13.^19,20^ In our study, the KRAS mutations were most commonly found in exon 2 (78.8%). Approximately three-fourths of these exon 2 mutations were codon 12, and one-fourth were codon 13 mutations, similar to the literature. Li et al^21^ reported that KRAS mutations in exon 2 codons 12 and 13 were most common in tumors with mucinous differentiation. In our study, KRAS exon 2 mutations were present in all 7 mucinous adenocarcinomas. While 6 of them were in codon 12 and 1 was in codon 13, the relationship between mucinous morphology and KRAS exon 2 mutations was statistically significant. Despite its increasing clinical importance, the clinicopathologic and molecular characteristics of CRCs with KRAS exon 3–codon 61 mutation and the prognostic and predictive values of this mutation are not yet sufficiently known. Various studies have reported this mutation’s rate as 1.3%-1.7%.^6,22^ In our study, KRAS exon 3 mutations were found at a higher rate (5.5%) compared to the literature, and 4.1% of these were exon 3–codon 61 mutations. We think that the positive correlation between KRAS exon 3 mutations and well-differentiated tumors in our study is valuable in detailing the relationship between this mutation and clinicopathologic parameters and will contribute to the literature on this subject for which there is no sufficient data yet.

The APC gene has been considered a tumor-suppressor gene in CRCs and is involved in the early stages of carcinogenesis. Even though APC is one of CRC’s most prevalently mutated driver genes, this gene is not included in prognostic classifications.^23^ In our study, we found 32.9% mutations in the APC gene, which is lower than the rate of mutations reported in the literature (50%-81%).^7^ Most of our patients were in the advanced stage, and this low rate may be due to regional determinants. The APC mutation, considered a potential target for carcinogenesis and targeted treatments in the literature and the prognostic significance of which was not determined in other studies, was also not associated with prognostic factors in our study. Chen et al^24^ reported that the interactions between MLH3, postmeiotic segregation increased1 homolog 2 (PMS2), and APC gene mutations enhanced tumor development and accelerated tumor progression. In our study, significant positive associations of APC mutations were present with MLH3, PTEN, CDC27, and BRCA2. It should be considered that APC, one of the genes showing significant association with some other mutations, might be effective through various pathways.

Phosphatase and tensin homolog (PTEN) mutation, mediated by genetic or epigenetic mechanisms, is detected in 20%-30% of CRCs, resulting in biallelic inactivation.^8^ Our study determined the PTEN mutation at a higher rate (32.9%) than the literature and found a significant correlation between stage pT2 tumors and the PTEN mutation. In addition, studies have reported that PTEN expression loss might be associated with the KRAS, BRAF, BRCA2, and APC mutations but that the mutations of PTEN and tumor protein 53 (TP53) were mutually exclusive.^8,25,26^ In our study, the associations of PTEN with BRCA2 and APC are of distinctive importance. The nuclear PTEN assists DNA repair by upregulating Rad51, an essential protein in repairing DNA double-strand breakages.^25^ Breast cancer 2 (BRCA2) is involved in the repair of DNA double-strand breaks through homologous recombination by forming the BRCA2/Rad51 complex.^27^ The significant association determined between the BRCA2 and PTEN mutations in our study is remarkable when these mechanisms are considered, because PTEN loss causes homologous recombination defects, which in turn sensitizes tumor cells against poly-ADP-ribose polymerase (PARP) inhibitors.^25^ Similarly, CRCs with BRCA2 mutations are known to be more sensitive to treatment with PARP inhibitors.^9^ Tabrizian et al^28^ reported in a mouse model that PTEN inactivation alone did not lead to tumor development even though it increased the proliferation rate in intestinal stem cells; on the other hand, PTEN inactivation potentiated tumor development from stem cells in the case of APC deficiency. Similarly, Marsh et al^26^ reported that tumor development and progression occurred rapidly in PTEN–APC loss. In this context, our study supports the effect of PTEN–APC mutation association on CRC carcinogenesis. In their study on using PI3K/mammalian Target of Rapamycin (mTOR) and mitogen-activated protein kinase (MEK) inhibitors as therapeutic targets in CRCs, Raja et al^29^ showed that PI3K/mTOR inhibition in CRCs is highly effective only in the presence of combined APC and PTEN mutations. Based on all these findings, PTEN may be a promising target for further studies both as a marker of progression and as an important indicator that can be utilized in treatment planning and predicting responses to therapeutics.

The PIK3CA mutation is reported in 12.7%-18.5% of CRCs and is highest in genomically stable cancers.^10^ Mucinous morphology in CRCs has been reported to be associated with the PIK3CA mutation.^30^ The incidence of PIK3CA mutation varies according to the anatomical site, with the highest rate found in cecal cancers (30%), 18% in non-rectal colon cancers, and 9% in rectal cancers.^10^ A significant association between the PIK3CA and KRAS mutations has also been documented.^30^ In our study, the frequency of PIK3CA mutation (24.6%) was higher than that in the literature, and its association with perineural invasion, one of the clinicopathologic parameters, was found to be significant. However, no literature data support this finding yet.

Serine/threonine protein kinase B-raf (BRAF) mutations are found in 8%-12% of all CRCs, and 90%-96% of these are BRAF V600E mutations.^11,31,32^ Two molecular subtypes with different clinicopathologic characteristics, the BRAF V600E and non-V600E BRAF mutations, have been described in CRCs.^32^ In general, both BRAF and BRAF V600E mutations have been found more frequently in CRCs of elderly and female patients located in the proximal colon and with poorly differentiated and mucinous morphology.^12,31^ The rates of 17.8% for BRAF mutations and 13.7% for BRAF V600E mutations determined in our study were relatively high compared to recent meta-analyses. On the other hand, various studies have reported that CRCs with BRAF mutations were more common in Caucasians compared to Asian or African-American individuals.^31^ The higher rates of BRAF and BRAF V600E mutations determined in our study suggest that the mutation rate might be associated with the ethnicity factor. This result is of importance for establishing a data pool for our population located between Europe and Asia. Furthermore, the association that our study determined between the BRAF mutation and poorly differentiated tumors has been strongly supported by meta-analysis data.^33^

Colorectal carcinoma, which is a heterogeneous disease, exhibits various molecular alterations.^34^ The mutations involved in CRC pathogenesis differ depending on proximal or distal colon tumor, and molecular analysis is a cornerstone in the treatment and prognosis of CRCs.^35^ There are 3 different molecular pathways identified so far in the pathogenesis of CRC: (i) chromosomal instability (CIN) (non-hypermutated pathway), (ii) microsatellite instability (MSI) (hypermutated pathway), and (iii) DNA polymerase proofreading mutations (ultramutant pathway). These pathways are not mutually exclusive, and some tumors show features of more than 1 pathway.^1,2^ The pathological and molecular features of proximal CRC differ significantly from distal CRC.^35^ Considering the relationship of molecular pathways with tumor localization, while proximal CRCs tend to be MSI high, distal CRCs are CIN-high tumors. KRAS, TP53, APC, PIK3CA, F-box and WD-40 domain-containing protein 7 (FBXW-7), Transcription factor 7 like 2 (TCF7L2), Sma- and Mad-associated protein 4 (SMAD4), and neuroblastom RAS viral (V-Ras) (NRAS) oncogene homolog are the most common mutations in distal CRCs, APC, Activin receptor type 2A (ACVR2A), MSH3, MSH6, transforming growth factor beta receptor 2 (TGFB-R2), TCF7L2, SLC9A9, and BRAF V600E in proximal CRCs.^7,34^ The significant association between the BRAF V600E mutation and tumors located in the proximal colon was the other finding of our study, which the literature data have also strongly supported.^11,31^ This finding reveals that molecular changes occurring in proximal and distal colon tumors have different clinical reflections.^33^ The prognoses of proximal and distal CRC are significantly different. Proximal colon tumors involve more aggressive molecular changes and have a poor prognosis. Accordingly, targeted therapies for proximal and distal CRCs are also different. Distal CRC patients benefit more from adjuvant chemotherapies and individualized treatments. Proximal CRC patients do not respond well to chemotherapies, but better treatment response is obtained with immunotherapies.^34,35^ Recognizing the pathological and molecular alterations between proximal and distal CRC is essential to the development of effective therapies and determining prognosis.

In CRCs, BRAF and KRAS mutations have been recognized as mutually exclusive. The KRAS mutation is a negative predictor for anti-EGFR treatment, and approximately 95% of patients with KRAS mutations do not respond to anti-EGFR treatment. Studies have suggested that the BRAF mutation has a potential negative predictive value for anti-EGFR treatment.^31^ The literature strongly supported our study’s significant negative association between BRAF and KRAS mutations.^33^ Patients with a non-BRAF V600E mutation have a better clinical course and more prolonged survival than those with a BRAF V600E mutation. These patients need less aggressive chemotherapeutics. For this reason, it is evident that the NGS method is essential for identifying undetectable BRAF mutations when the PCR tests established for the only BRAF V600E mutation are used.^36^

Tumor diameter has been known as one of the parameters with a controversial prognostic value in CRCs. Various studies have reported that the tumor diameter affected the overall patient survival.^37,38^ Moreover, some studies have reported that increased tumor diameter was associated with clinical parameters such as higher lymph node metastasis rate and more advanced T stage.^39^ Besides, CRCs with a higher rate of faulty mismatch repair (MMR) mechanisms have been reported to be associated with larger tumor diameter.^40^ Since the mutations in the MSH3 gene, 1 of the 7 MMR genes in humans, has been suggested to play a role in tumor progression in CRCs, studies on this subject have reported that loss of MSH3 expression was associated with lymph node metastasis and distant metastasis in CRCs.^41^ Our study revealed a significant association between the MSH3 mutation, for the prognostic value of which no consensus has yet been reached, and tumor diameter in CRCs. Since few studies have evaluated the clinicopathologic features of the MSH3 gene, no literature data support this finding. On the other hand, our study’s result is suggested to contribute to the literature regarding the association of the MSH3 gene’s mutations’ with poor prognosis.

Even though BRCA2, a tumor-suppressor gene that controls genome integrity, has a strong association with breast and ovarian cancers, the results regarding its association with CRC were contradictory.^42^ Zhunussova et al^43^ reported that the BRCA2 and APC genes were among the most frequently mutated tumor-suppressor genes. Pearlman et al,^44^ in their study analyzing genes predisposing to early-onset CRCs, showed a high prevalence of mutations in the BRCA2 gene. In our study, the BRCA2 mutation was found to be one of the mutations with a higher prevalence compared to the literature, and detecting such molecular alterations, which are not common, is essential in designing new individual treatment options. Because it is well known that patients having tumors with breast cancer 1 (BRCA1)/BRCA2 somatic mutations or loss benefit from PARP inhibitors, patients with mutations of these 2 tumor-suppressor genes might be therapeutic targets to be investigated for treatment, as demonstrated in our study.^9^ Despite the negative association found in our study between BRCA2 mutations and lymph node metastasis, which is a poor prognostic indicator, Xie et al^45^ reported a higher rate of mutations in the BRCA1/2 gene in CRCs with lymph node metastasis. Grabsch et al^46^ found no association between BRCA1/2 and lymph node metastasis. We believe that the results of our study on BRCA2, the relationship of which with lymph node metastasis has not been agreed upon and the association with clinicopathologic parameters has not yet been sufficiently investigated, will make a significant contribution to the literature.

Another point worth mentioning based on the genes included in our study is the relationship between repair pathways of DNA double-strand breaks and MSI. Unfortunately, even though MMR genes such as MLH3, MSH2, MSH3, and MSH6 were included in our study, MSI was not studied. However, determining MSI status in CRCs and mutations in the genes involved in repairing DNA double-strand breaks such as BRCA1/2 and uncovering the associations of these pathways with each other is essential in the context of treatment, because it is suggested that CRCs with MSI intact pathways for repairing DNA double-chain breaks are more sensitive to chemotherapeutics that act by generating double-chain breaks in DNA. For this reason, further studies are needed to determine a functional link between MMR mechanisms and DNA double-strand break reparation pathways and to suggest that these pathways are interrelated.^46^

## Conclusion

When compared to the literature, the CDC27, KRAS, BRAF, BRAF, PTEN, PIK3CA, and BRCA2 mutations were determined at a higher rate and the APC mutation at a lower rate in the somatic mutation profile identified by the NGS system in patients with CRC in a specific region of the Turkish population. Furthermore, the significant positive correlations between CDC27 and PTEN, PTEN and BRCA2, and PTEN and APC mutation associations and the significant negative association between the BRAF and KRAS mutations were of significant importance regarding tumor development and progression and increased resistance or sensitivity against treatments. Significant relationships determined between KRAS exon 2 and mucinous morphology, PIK3CA and absence of perineural invasion, BRAF and tumor differentiation and localization, MSH3 and tumor diameter, and BRCA2 and absence of lymph node metastasis were findings that have contributed to the literature. Despite the relatively small number of patients, our study’s results may bring different perspectives for new studies. By revealing regional and racial somatic mutation differences, it will be easier to determine new therapeutic and prognostic criteria in CRC patients. As the molecular mechanisms leading to CRC development, progression, and metastasis are better elucidated, the importance of biomarkers that may be associated with disease outcome, metastasis, or treatment resistance will increase.

## Figures and Tables

**Table 1.** Gene Content of Colorectal Cancer Panel (DHS-002Z-12)

AKT1	CDC27	KRAS	PIK3CA
APC	CDK4	MET	PMS2
ATM	DCC	MLH1	PTEN
BAX	EGFR	MLH3	RET
BRAF	EPCAM	MSH2	SMAD2
BRCA1	ERBB2	MSH3	SMAD4
BRCA2	FBXW7	MSH6	TGFBR2
CASP8	KIT	NRAS	TP53

AKT1, V-Akt murine thymoma viral oncogene homolog 1; APC, adenomatous polyposis coli; ATM, ataxia telangiectasia mutant; BAX, B-cell lymphoma 2-associated X; BRAF, serine/threonine protein kinase B-raf; BRCA1, breast cancer 1; BRCA2, breast cancer 2; CASP8, caspase 8; CDC27, cell division cycle 27; CDK4, cyclin-dependent kinase 4; DCC, deleted in colorectal cancer; EGFR, epidermal growth factor receptor; EPCAM, epithelial cell adhesion molecule; ERBB2, erythroblastic oncogene B 2; FBXW7, F-box and WD-40 domain-containing protein 7; KIT, V-Kit Hardy–Zuckerman 4 feline sarcoma viral oncogene homolog; KRAS, Kirsten rat sarcoma viral proto-oncogene; MET, mesenchymal–epithelial transition tyrosine kinase; MLH1, MutL homolog 1; MLH3, MutL homolog 3; MSH2, MutS homolog 2; MSH3, MutS homolog 3; MSH6, MutS homolog 6; NRAS, neuroblastom RAS viral (V-Ras) oncogene homolog; PIK3CA, phosphotidylinositol-4,5-biphosphate 3-kinase; PMS2, postmeiotic segregation increased1 homolog 2; PTEN, phosphatase and tensin homolog; RET, rearranged during transfection; SMAD2, Sma- and Mad-associated protein 2; SMAD4, Sma- and Mad-associated protein 4; TGFBR2, transforming growth factor beta receptor 2; TP53, tumor protein 53.

**Table 2.** Clinicopathological Features and Distribution of Patients

Clinicopathologic Features	
Sex, n (%)	
Female	26 (35.6)
Male	47 (64.4)
Age, years	
Age range	32-86
Mean age, mean ± SD	62.53 ± 11.126
Tumor localization, n (%)	
Proximal colon	27 (37)
Distal colon	29 (39.7)
Rectum	17 (23.3)
Tumor diameter (cm)	
Diameter range	1.5-11.5
Mean diameter	4.5 ± 1.9230
Tumor differentiation, n (%)	
Well differentiated	11 (15.1)
Moderately differentiated	46 (63)
Low differentiated	7 (9.6)
Mucinous	9 (12.3)
Pathological stage, n (%)	
pT1	1 (1.4)
pT2	9 (12.3)
pT3	51 (69.9)
pT4	12 (16.4)
Lymphovascular invasion, n (%)	
Available	63 (86.3)
Absent	10 (13.7)
Perineural invasion, n (%)	
Available	51 (69.9)
Absent	22 (30.1)
Lymph node metastasis, n (%)	
Available	39 (53.4)
Absent	34 (46.6)

**Table 3.** Concomitant Mutations Detected in the Patients

Concomitant Mutations	n (%)	Concomitant Mutations	n (%)
CDC27–PTEN	22 (30.13)	BRCA1–CDC27	5 (6.8)
APC–CDC27	21 (28.8)	BRCA1–TGFBR2	5 (6.8)
APC–PTEN	17 (23.2)	CDC27–FBXW7	5 (6.8)
CDC27–MLH3	13 (17.2)	BAX–CDC27	4 (5.5)
CDC27–MSH3	13 (17.2)	BRCA1–BRCA2	4 (5.5)
BRCA2–CDC27	11 (15)	BRCA1–MLH3	4 (5.5)
MLH3–PTEN	11 (15)	BRCA1–MSH3	4 (5.5)
MSH3–PTEN	11 (15)	FBXW7–PTEN	4 (5.5)
CDC27–TGFBR2	10 (13.7)	CDC27–PIK3CA	3 (4.1)
APC–MLH3	9 (12.3)	FBXW7–PMS2	3 (4.1)
MLH3–MSH3	9 (12.3)	FBXW7–TGFBR2	3 (4.1)
APC–BRCA2	8 (11)	BAX–BRCA1	2 (2.7)
BRCA2–PTEN	8 (11)	BRCA1–MSH6	2 (2.7)
CDC27–PMS2	8 (11)	FBXW7–MSH6	2 (2.7)
MSH3–TGFBR2	8 (11)	MSH3–SMAD2	2 (2.7)
PTEN–TGFBR2	8 (11)	MSH6–TGFBR2	2 (2.7)
APC–TP53	7 (9.6)	PIK3CA–PTEN	2 (2.7)
APC–TGFBR2	7 (9.6)	MSH6–TGFBR2	2 (2.7)
MLH3–TGFBR2	7 (9.6)	SMAD2–TGFBR2	2 (2.7)
PMS2–PTEN	7 (9.6)	BRCA1–MSH2	1 (1.4)
APC–PMS2	6 (8.2)	EGFR–ERBB2	1 (1.4)
BRCA2–MLH3	6 (8.2)	BRAF–KRAS	0 (0)

APC, adenomatous polyposis coli; BAX, B-cell lymphoma 2-associated X; BRAF, serine/threonine protein kinase B-raf; BRCA1, breast cancer 1; BRCA2, breast cancer 2; CDC27, cell division cycle 27; EGFR, epidermal growth factor receptor; ERBB2, erythroblastic oncogene B 2; FBXW7, F-box and WD-40 domain-containing protein 7; KRAS, Kirsten rat sarcoma viral proto-oncogene; MLH1, MutL homolog 1; MLH3, MutL homolog 3; MSH2, MutS homolog 2; MSH3, MutS homolog 3; MSH6, MutS homolog 6; PIK3CA, phosphotidylinositol-4,5-biphosphate 3-kinase; PMS2, postmeiotic segregation increased1 homolog 2; PTEN, phosphatase and tensin homolog; SMAD2, Sma- and Mad-associated protein 2; TGFBR2, transforming growth factor beta receptor 2; TP53, tumor protein 53.

**Table 4.** Comparison of Mutations Detected in the Current Study with International and National Literature

Mutations	International Literature (%)	National Literature (%)	
		Previous Studies	Current Study
AKT1	0.5-1.7	—	—
APC	51-81	—	**32.9**
ATM	7	—	6.8
BAX	2.1	—	5.5
BRAF	8-12	2-33.6	**17.8**
BRCA1	0.3-2.6	—	6.8
BRCA2	0.34-2	—	**16.4**
CASP8	5.1	—	—
CDC27	5	—	**46.6**
CDK4	0.86	—	—
DCC	6	—	—
EGFR	0.9-22.4	—	2.7
EPCAM	—	—	—
ERBB2	2.8-8.3	—	1.4
FBXW7	7.3-20	—	6.8
KIT	0.3-2.8	—	—
KRAS	30-40	30-49	**45.2**
MET	0-9.5	—	—
MLH1	0.9-77	—	—
MLH3	8.6-25	—	**17.8**
MSH2	0.9-40	—	1.4
MSH3	3.8-59.3	—	**17.8**
MSH6	0.4-40	—	4.1
NRAS	2.9-8	3.2-17.9	1.4
PIK3CA	12.7-20.8	—	**24.7**
PMS2	0.6-3.1	—	11
PTEN	0.6-20	—	**32.9**
RET	0.17-2.7	—	—
SMAD2	0-10	—	2.7
SMAD4	2-15	—	8.2
TGFBR2	3.1-72	—	13.7
TP53	6-60	—	11

AKT1, V-Akt murine thymoma viral oncogene homolog 1; APC, adenomatous polyposis coli; ATM, ataxia telangiectasia mutant; BAX, B-cell lymphoma 2-associated X; BRAF, serine/threonine protein kinase B-raf; BRCA1, breast cancer 1; BRCA2, breast cancer 2; CASP8, caspase 8; CDC27, cell division cycle 27; CDK4, cyclin-dependent kinase 4; DCC, deleted in colorectal cancer; EGFR, epidermal growth factor receptor; EPCAM, epithelial cell adhesion molecule; ERBB2, erythroblastic oncogene B 2; FBXW7, F-box and WD-40 domain-containing protein 7; KIT, V-Kit Hardy–Zuckerman 4 feline sarcoma viral oncogene homolog; KRAS, Kirsten rat sarcoma viral proto-oncogene; MET, mesenchymal–epithelial transition tyrosine kinase; MLH1, MutL homolog 1; MLH3, MutL homolog 3; MSH2, MutS homolog 2; MSH3, MutS homolog 3; MSH6, MutS homolog 6; NRAS, neuroblastom RAS viral (V-Ras) oncogene homolog; PIK3CA, phosphotidylinositol-4,5-biphosphate 3-kinase; PMS2, postmeiotic segregation increased1 homolog 2; PTEN, phosphatase and tensin homolog; RET, rearranged during transfection; SMAD2, Sma- and Mad-associated protein 2; SMAD4, Sma- and Mad-associated protein 4; TGFBR2, transforming growth factor beta receptor 2; TP53, tumor protein 53.
